# Prognostic Value of Various Hemostasis Parameters and Neurophysiological Examinations in Spontaneous Intracerebral Hemorrhage: The IRONHEART Study Protocol

**DOI:** 10.3389/fneur.2021.615177

**Published:** 2021-03-17

**Authors:** Tamás Árokszállási, Máté Héja, Zsuzsa Bagoly, Kitti Bernadett Kovács, Rita Orbán-Kálmándi, Ferenc Sarkady, Judit Tóth, Klára Fekete, István Fekete, László Csiba

**Affiliations:** ^1^Department of Neurology, Faculty of Medicine, University of Debrecen, Debrecen, Hungary; ^2^Magyar Tudományos Akadémia-Debreceni Egyetem (MTA-DE) Cerebrovascular and Neurodegenerative Research Group, University of Debrecen, Debrecen, Hungary; ^3^Division of Clinical Laboratory Sciences, Department of Laboratory Medicine, Faculty of Medicine, University of Debrecen, Debrecen, Hungary; ^4^Department of Radiology, Faculty of Medicine, University of Debrecen, Debrecen, Hungary

**Keywords:** intracerebral hemorrhage, outcome, clot lysis, motor evoked potential, quantitative electroencephalography

## Abstract

**Rationale:** Stroke is one of the leading causes of death in all developed countries. In Hungary, more than 10,000 patients die annually due to cerebrovascular diseases according to the WHO Mortality Database. Of these patients, 10–15 % suffer non-traumatic intracerebral hemorrhage (ICH). ICH results in a higher rate of mortality as compared to ischemic stroke and outcomes are difficult to predict. In the IRONHEART study, we aim to test various hemostasis parameters and clinical neurophysiological examinations in evaluating outcome in ICH.

**Methods:** In this prospective, observational study, we plan to enroll consecutive patients with non-traumatic spontaneous ICH admitted to a single Stroke Center (Department of Neurology, University of Debrecen, Hungary). The protocol of the IRONHEART study includes the investigation of detailed clinical, laboratory investigations, and various neurophysiological examinations. Stroke severity is quantified based on the National Institutes of Health Stroke Scale (NIHSS) on admission and day 7, 14, and 90 after the onset of stroke. Cranial CT is performed on admission, day 14, and 90 to estimate the ICH volume. Modified Rankin Scale (mRS) is used for evaluating the long-term outcome (90 days post-event). Blood is drawn immediately on admission for specific hemostasis tests. Digital and quantitative EEG techniques and motor evoked potential (MEP) are performed to evaluate the prognosis of cerebral hemorrhage on admission (within 24–48 h), immediately before discharge (~10–14 days later), and 3 months after the event.

**Outcomes:** The following outcomes are investigated: primary outcomes: mortality by day 14 and day 90, secondary long-term outcome at 90 days post-event where mRS 0–2 is defined as favorable long-term outcome.

**Discussion:** If associations between outcomes and the investigated parameters (hemostasis and neurophysiological examinations) are confirmed, results might aid prognosis assessment in this subtype of stroke with particularly high mortality. Improving clinical grading systems on ICH severity and outcomes by including the investigated parameters could help to better guide the management of these patients in the future.

## Introduction

Stroke is one of the leading causes of death in all developed countries (1). In Hungary, more than 10,000 patients die annually due to cerebrovascular diseases according to the WHO Mortality Database (2). Of these patients, 10–15 % suffer non-traumatic intracerebral hemorrhage (ICH), which has the highest case fatality among all strokes (3). Age over 80 years, infrantentorial origin, intraventricular hemorrhage, hemorrhage volume ≥30 cm^3^, Glasgow Coma Scale score under 5 are particularly poor prognostic factors (4). Although its mortality is higher than that of ischemic stroke, no similar therapeutic options are available. This might be explained by the fact that its etiology often remains unknown and outcomes in ICH are difficult to predict. In the IRONHEART study, detailed clinical, laboratory investigations, and the results of various neurophysiological examinations will be correlated with ICH stroke severity and clinical outcomes.

Despite the significant mortality of ICH and the considerable health care burden related to treatment and rehabilitation of ICH patients, management strategies have not improved significantly in the past decade. This is at least partly due to an existing knowledge gap of the pathophysiology of the intracerebral bleeding and mechanisms driving hematoma progression. In the IRONHEART study, we hypothesize that by learning more about the levels of hemostasis factors and the fibrinolytic potential in ICH patients and their relation to clinical outcomes we might better understand the underlying pathomechanism that could be the key for improved therapeutic approaches in the future. Neurophysiological examinations might equally have considerable potentials as prognostic tools in predicting changes in the extent of bleeding and brain edema. Here we hypothesize that identification of the above parameters employing neurophysiological examinations could be important because they could have a significant impact on the outcome of brain hemorrhage and might provide an early tool for the personalization of ICH treatment.

The main goals of the hemostasis substudy are the following: (1) to examine the levels of certain coagulation and fibrinolysis markers in ICH patients and correlate results with outcomes and cerebral hematoma volume (2) to introduce a new, modified global assay of clot formation and fibrinolysis that includes the effect of NETs and to test its potential clinical utility in ICH patients.

The main goals of the neurophysiological substudy are the following: (1) to find out whether quantitative EEG results correlate with the improvement or deterioration of cerebral functions by comparing data with clinical parameters (NIHSS score, modified Rankin Scale) and cranial CT findings (estimated hematoma and edema volume). (2) Early detection of epileptiform discharges or non-convulsive seizures to select patients who may benefit from early-initiated antiseizure drug treatment. Parallel with the quantitative EEG we will perform TMS examinations to evaluate the prognosis and the probable effectiveness of further rehabilitation. The main purpose is to look for a correlation between intracerebral blood volume, magnetic evoked potential, and functional outcome.

## Methods and Analysis

### Patients

**Figure 1:**
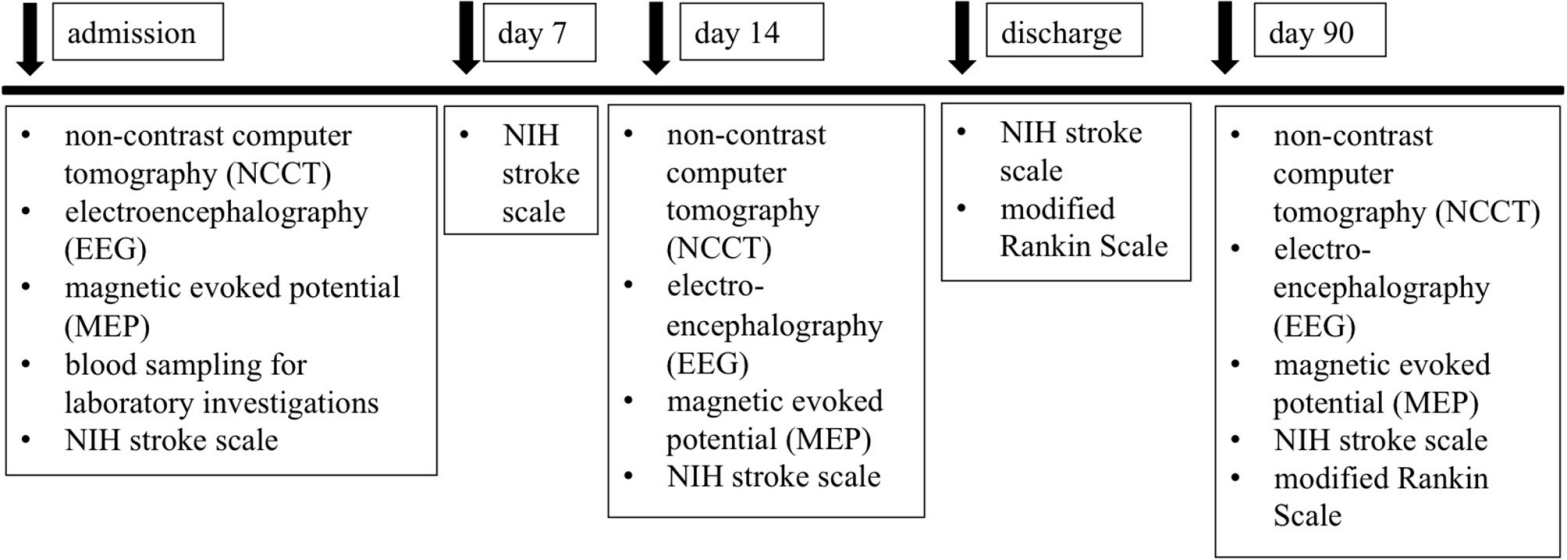
Flow chart of examinations planned during the study.

In this prospective, observational study, we plan to enroll consecutive patients with ICH admitted to a single Stroke Center (Department of Neurology, University of Debrecen, Hungary). Inclusion criteria: patients over 18 years with acute non-traumatic, spontaneous ICH verified with cranial CT, not meeting exclusion criteria. Exclusion criteria: the presence of cerebral aneurysm, arteriovenous malformation, malignancy, subdural and epidural hemorrhage, severe hepatic- or renal insufficiency, hemorrhagic diathesis. A detailed flowchart of examinations is depicted in Figure 1. ICH will be diagnosed with complex physical examination and non-contrast computerized tomography (NCCT) scan on arrival. If necessary, contrast-enhanced CT or CT-angiography is performed on admission to rule out secondary causes of ICH (e.g., aneurysm, cerebral tumors). Follow-up NCCT scans will be performed 14 ± 2 days and 3 months ± 7 days after the stroke onset. CT images will be analyzed simultaneously by 3 independent radiologists, and a detailed list of radiological data, such as location and volume of bleeding, extent of perihematomal edema, degree of midline shift, involvement of pyramidal tract, and presence of intraventricular or subarachnoidal component will be recorded (5). The volume of the IVH will be not measured. Manual CT volumetry will be performed by tracing the focal hyperdense (blood) the and the perifocal hypodense area surrounding the hemorrhage (edema) on each slice. Hypodensity attributable to microangiopathy will be omitted as far as possible by comparison with the contralateral hemisphere. Total lesion volume will be calculated by multiplying the traced area (ROI, cm^2^) with the slice thickness and adding up the results (6–8). All tracings will be performed by one radiologist.

The time of symptom onset, baseline characteristics (age, sex, BMI, cerebrovascular risk factors, history of cerebrovascular and cardiovascular diseases, previous medications) will be recorded on admission. National Institutes of Health Stroke Scale (NIHSS) will be used to quantify stroke severity on admission and day 7 after the onset of hemorrhage stroke (9). Modified Rankin Scale (mRS) will be used for evaluating long-term outcomes (90 days post-event) (10) performed by neurologists of the study including physical examination. Mortality by day 14 and day 90 will be assessed. All patients who die during the hospital stay will be autopsied and the cause of death will be assessed and determined by a pathologist. When autopsy is performed amyloid angiopathy and microbleeding will be examined. Cerebral microbleeds may help to predict the prognosis of ICH (11).

### Outcomes

The following primary outcomes are defined: 1/ Mortality by day 14 and day 90 2/Long-term outcome at 90 days post-event: mRS 0–2 is defined as favorable long-term outcome.

### Informed Consent

The study design was developed by the guiding principles of the Declaration of Helsinki and was approved by the Institutional Ethics Committee of the University of Debrecen and the Ethics Committee of the National Medical Research Council. All patients or their relatives are required to provide written informed consent to be enrolled in the study. A general consent form encompasses the consent for blood sampling, imaging studies, electrophysiological examinations, and autopsy also. All patient data will be treated anonymously.

### Blood Sampling and Laboratory Measurements

Peripheral venous blood samples will be taken from all enrolled patients on admission (within 1 h of admittance). Routine laboratory tests (ions, glucose level, renal and liver function tests, high-sensitivity C-reactive protein measurement, complete blood count) will be carried out immediately after blood drawing by standard laboratory methods (Roche Diagnostics, Mannheim, Germany, and Sysmex Europe GmbH, Hamburg, Germany). For the examination of hemostasis tests, blood samples will be collected to vacutainer tubes containing 0.109 M sodium citrate (Becton Dickinson, Franklin Lane, NJ) and plasma samples will be processed immediately (centrifugation twice at 1,500 g, room temperature, 15 min). Screening tests of coagulation (prothrombin time, activated partial thromboplastin time, and thrombin time) will be carried out immediately using standard methods (BCS coagulometer, Siemens Healthcare Diagnostic Products, Marburg, Germany) Plasma samples will be labeled with a code and stored at −80 C until further analysis of specific hemostasis measurements. All measurements will be carried out by investigators blinded to clinical data. Quantitative D-dimer levels will be measured by a particle-enhanced, immuno-turbidimetric assay (Innovance D-dimer) on a BCS coagulometer according to the manufacturer's instructions (Siemens Healthcare Diagnostic Products, Marburg, Germany). The levels of von Willebrand factor (VWF) antigen, chromogenic factor VIII (FVIII) activity, α2-plasmin inhibitor (α2-PI) activity, and plasminogen activity will be measured by commercially available methods (Siemens Healthcare Diagnostic Products, Marburg, Germany). Fibrinogen levels will be analyzed according to the method of Clauss. Plasma levels of factor FXIII (FXIII) activity will be determined by ammonia release assay using a commercially available reagent kit (REA-chrom FXIII kit, Reanalker, Budapest, Hungary). PAI-1 activity and antigen levels will be measured using commercially available ELISA tests (Technozym PAI-1 Actibind ELISA and Technozym PAI-1 Antigen ELISA assays, respectively), according to the manufacturer's instructions. Thrombin generation assay will be performed using the Thrombinoscope CAT (Calibrated Automated Thrombogram, Maastricht, The Netherlands) assay according to the manufacturer's instructions (Diagnostica Stago, Asnières, France).

### Neurophysiological Examinations

As a part of this study, we will perform digital and quantitative EEG and MEP examinations on all enrolled participants. Patients will be examined on 3 occasions with the above neurophysiological examinations within 24–48 h after admission, at 14 days ± 2 days and 3 months ± 7 days after the event. The EEG and MEP will be analyzed by clinical neurophysiologists participating in the study (KF and IF). EEGs will be recorded in the EEG laboratory of the Department of Neurology according to the International Federation of Clinical Neurophysiology guidelines using Micromed BrainQuick Plus System with surface Ag/AgCl electrodes. Each recording session will last for 40 min. There are no exclusion criteria for digital and quantitative EEG. Monophasic, single-pulse stimulation is used by Magstim 200 magnetic stimulator. Central motor conduction time and amplitudes will be recorded and analyzed. Patients would be selected after strict consideration of contraindications (e.g., metallic implantations, pacemaker are excluding factors) as described in safety regulations of MEP examination protocol. Recording electrodes will be placed over the abductor digiti minimi muscle of the upper limb, and on the anterior tibial muscle of the lower limb. The investigation is suitable to show the severity of corticospinal tract damage which may correlate with the prognosis and outcome of rehabilitation even in a hemiplegic patient (12).

### Sample Size Calculations and Statistical Analysis

Sample size calculations were performed using the power/sample size calculator software of the Department of Biostatistics, Vanderbilt University. Based on our calculations using the Casagrande, Pike and Smith's method, assuming a 15% true difference between the compared groups based on primary and secondary outcomes, an estimated sample size of 87 patients was determined by setting the value of α to 0.05 and the statistical power to 0.80 (in case of at least 30% anticipated incidence of outcome event, at least 25 patients in each outcome group should be enrolled plus 6% additional subjects for attrition). Patient groups will be dichotomized according to mortality by day 14 and day 90, and to groups according to favorable (mRS 0–2) or unfavorable outcomes (mRS 3–6). According to previous records, this sample size can be reached in our center in a period of ~4 years. For the statistical evaluation of the results, the Statistical Package for Social Sciences (SPSS, Version 26.0, Chicago, IL), and GraphPad Prism 8.0 (GraphPad Prism Inc., La Jolla, CA) software will be used. The normality of data will be studied using the Shapiro-Wilk test. Student's *t*-test or Mann–Whitney *U*-test will be performed for independent two-group analyses. In the case of paired data, paired *t*-test or Wilcoxon signed-rank test will be applied. ANOVA with Bonferroni *post-hoc* test or Kruskal–Wallis analysis with Dunn's *post-hoc* test will be used for multiple comparisons. Pearson's or Spearman's correlation will be used to determine the strength of association between continuous variables. Differences between categorical variables will be assessed by χ^2^ test or by Fisher's exact where appropriate. In the case of hemostasis variables, optimal tests and threshold values will be chosen using receiver operating characteristic (ROC) curves, built by plotting sensitivity vs. 1-specificity, calculating the area under the curve (AUC), and defining threshold values based on Youden's J statistics. Test characteristics of sensitivity, specificity, positive predictive value (PPV), and negative predictive value (NPV) will be calculated using contingency tables and χ^2^ test or Fisher's exact at statistically optimal threshold values. The Kaplan-Meier method will be applied to plot survival vs. non-survival of patients, based on previously calculated optimal test parameter cut-offs. Survival curves will be compared using the log-rank test.

## Discussion

Non-traumatic, spontaneous ICH is one of the most disabling forms of stroke, with high mortality and limited therapeutic choices. To make treatment decisions and being able to determine a prognosis, it is important to know which factors predict outcome. Most studies in this field investigate clinical and radiographic predictors of ICH outcome, e.g., age, premorbid functional state, initial GCS, blood pressure, hematoma location, and volume (13, 14). Non-invasive neuromonitoring techniques, such as quantitative EEG and evoked potentials, are increasingly being used to monitor brain damage in traumatic brain injury and subarachnoidal hemorrhage to evaluate functional recovery (15, 16), but only a few studies deal with primary ICHs. As this field of stroke research is relatively understudied, new data is warranted by studying relatively large prospective patient cohorts to understand the pathophysiology and the progression of the disease.

Few studies showed that coagulation disorders and impaired hemostasis increase the risk of ICH (17, 18) but the impact of hemostasis and fibrinolytic abnormalities on the outcome of non-traumatic ICH has not been thoroughly evaluated. Besides a handful of studies indicating that increased admission D-dimer levels are associated with ICH mortality (19–21), the role of hemostasis or fibrinolytic system alterations potentially occurring in ICH has remained mostly unexplored in predicting outcomes. Gaining knowledge on hemostasis factors that potentially drive the enlargement or the dissolution of the ICH might be crucial for future therapeutic approaches. As a first step, the underlying pathomechanism leading to poor outcomes must be understood in patients, and adequate diagnostic tools are substantial for this. It is important to learn about the levels of certain individual hemostasis and fibrinolytic factors in ICH patients as they might play a role in the development and dissolution of the hematoma. However, a global assay of coagulation and fibrinolysis might prove to be more useful in the acute clinical setting as compared to laborious individual tests. The clot lysis assay (CLA) is a global assay of clot formation and fibrinolysis that provides valuable information about the fibrinolytic potential of the plasma. This measurement is performed using plasma samples therefore the cellular components of hemostasis are not investigated by this test. In the past decade, it has been reported that neutrophil extracellular trap (NET) components are important modulating factors of hemostasis: NET components intercalate to fibrin and alter clot formation and fibrinolysis (22, 23). The prothrombotic and antifibrinolytic effects of NETs may play a role in the clinical outcome of ICH. To our knowledge, our study is the first that plans to examine whether a modified CLA that incorporates the effects of NETs might be a prognostic factor in patients with non-traumatic ICH.

Neurophysiological examinations might also have tremendous potentials in predicting ICH outcomes, however, the prognostic value of EEG and MEP in ICH outcomes has not been extensively studied. Changes in the extent of bleeding, moreover, the onset and progression of brain edema can be monitored by using digital and quantitative EEG techniques (24). During early management, identification of these parameters is important because it could have a significant impact on the outcome of brain hemorrhage. In the early stages, localized polymorphic delta wave activity appears ipsilaterally in patients with ICH without a shift of midline structures, regardless of the location of hematoma. In patients with larger hematomas of 30 mL or more, causing a shift of the midline structures, delta wave activity appears over both hemispheres, while superposition of faster frequencies on slow waves indicates the development of brain edema (25). The incidence of seizures after spontaneous ICH reportedly ranges from 2.8 to 18.7% (26). Provoking factors are related to hemorrhage volume, hemorrhage location within the cerebrum, cortical involvement, and the severity of neurological deficits (12). Seizures can be non-convulsive, which remain undiscovered without an EEG examination. The incidence of non-convulsive seizures has been reported in 8–20% of ICU patients, and in case it progresses to status epilepticus, the mortality could be as high as 70% (27). Epileptiform discharges detected by EEG or early seizures are associated with deterioration of mental functions and should be treated with antiepileptic drugs (28). There is a strong relationship between the pathophysiology of ICH and the development of epilepsy. Therefore, early antiepileptic treatment has a positive effect on glutaminergic synaptic transmission, neuronal cell apoptosis, and permeability of the blood-brain barrier through decreasing inflammatory processes (29). Damage to the corticospinal tract can be examined with motor evoked potentials (MEP) elicited by transcranial magnetic stimulation (TMS). Based on the literature, the absence of MEP in the acute stage indicates poor recovery of muscle strength, while the presence of MEP in a completely hemiplegic patient predicted some recovery of motor function (12). The suppression of amplitude was more accurate than the prolongation of latency in predicting functional recovery. MEP monitoring of patients with hypertensive ICH in the acute stage can predict the outcome of motor function (30–32).

The IRONHEART study is a single-centered observational study, with the disadvantage of somewhat limited sample size, but with the clear advantage of uniform patient management, uniform neurophysiological examination, blood sample handling, and testing, which are crucial for the evaluation of results. In multicentric studies, specific investigations, including neurophysiological and hemostasis tests are difficult to harmonize due to the lack of standardized methods and due to the various sensitivity and specificity of the tests used. In the IRONHEART study, a complex evaluation of ICH progression will be carried out, and associations with specific hemostasis test results, neurophysiological data, and imaging data will be investigated. If associations between outcomes and the investigated parameters are confirmed, results might aid prognosis assessment in this subtype of stroke, with particularly high mortality. In our study, patients who die during the hospital stay will be autopsied, which will provide the possibility to differentiate between hemorrhagic stroke-related death and non-stroke related mortality as an outcome. Gaining knowledge on factors that potentially drive the enlargement or the dissolution of the ICH might be crucial for future therapeutic approaches. In the IRONHEART study, ICH size and its subsequent resolution or enlargement will be followed in every patient, providing potentially useful data in this respect. In the future, improving clinical grading systems on ICH outcomes by including the investigated parameters of the IRONHEART study might help to better guide the management of these patients. The MEP examinations will show us the proper time to evaluate the prognosis and will show the best biological marker and the appropriate parameter. The findings of the study will help clinicians estimate long-term outcome by using functional examinations such as EEG and MEP, instead of using imaging techniques only. Clinical neurophysiological examinations and CT findings together help planning the rehabilitation program tailored to the patients' status. The early epileptiform activity could predict early and delayed epileptic seizures, and presumably, early treatment might lead to more favorable outcome of the stroke.

## Ethics Statement

The study design was built by the guiding principles of the Declaration of Helsinki and was approved by the Institutional Ethics Committee of the University of Debrecen and the Ethics Committee of the National Medical Research Council (Registration Number: 16343-5/2017/EÜIG). All patients or their relatives will provide written informed consent to be enrolled in the study.

## Author Contributions

All authors listed have made a substantial, direct and intellectual contribution to the work, and approved it for publication.

## Conflict of Interest

The authors declare that the research was conducted in the absence of any commercial or financial relationships that could be construed as a potential conflict of interest.

## References

[1] Feigin VL. Stroke in developing countries: can the epidemic be stopped and outcomes improved? Lancet Neurol. (2007) 6:94–7. 10.1016/S1474-4422(07)70007-8 17239789

[2] Hartley A Marshall DC Salciccioli JD Sikkel MB Maruthappu M Shalhoub J. Trends in mortality from ischemic heart disease and cerebrovascular disease in Europe: 1980 to 2009. Circulation. (2016) 133:1916–26. 10.1161/CIRCULATIONAHA.115.018931 27006480

[3] Feigin VL Lawes CM Bennett DA Barker-Collo SL Parag V. Worldwide stroke incidence and early case fatality reported in 56 population-based studies: a systematic review. Lancet Neurol. (2009) 8:355–69. 10.1016/S1474-4422(09)70025-0 19233729

[4] Hemphill JC 3rd Bonovich DC Besmertis L Manley GT Johnston SC. The ICH score: a simple, reliable grading scale for intracerebral hemorrhage. Stroke. (2001) 32:891–7. 10.1161/01.STR.32.4.891 11283388

[5] Kothari RU Brott T Broderick JP Barsan WG Sauerbeck LR Zuccarello M. The ABCs of measuring intracerebral hemorrhage volumes. Stroke. (1996) 27:1304–5. 10.1161/01.STR.27.8.1304 8711791

[6] Al-Mufti F Thabet AM Singh T El-Ghanem M Amuluru K Gandhi CD. Clinical and radiographic predictors of intracerebral hemorrhage outcome. Interv Neurol. (2018) 7:118–36. 10.1159/000484571 29628951 PMC5881146

[7] Vinciguerra L Bosel J. Noninvasive neuromonitoring: current utility in subarachnoid hemorrhage, traumatic brain injury, and stroke. Neurocrit Care. (2017) 27:122–40. 10.1007/s12028-016-0361-8 28004334

[8] Volbers B Staykov D Wagner I Dorfler A Saake M Schwab S. Semi-automatic volumetric assessment of perihemorrhagic edema with computed tomography. Eur J Neurol. (2011) 18:1323–8. 10.1111/j.1468-1331.2011.03395.x 21457176

[9] Brott T Adams HP Jr Olinger CP Marler JR Barsan WG. Measurements of acute cerebral infarction: a clinical examination scale. Stroke. (1989) 20:864–70. 10.1161/01.STR.20.7.864 2749846

[10] van Swieten JC Koudstaal PJ Visser MC Schouten HJ van Gijn J. Interobserver agreement for the assessment of handicap in stroke patients. Stroke. (1988) 19:604–7. 10.1161/01.STR.19.5.604 3363593

[11] Charidimou A Imaizumi T Moulin S Biffi A Samarasekera N Yakushiji Y. Brain hemorrhage recurrence, small vessel disease type, and cerebral microbleeds: A meta-analysis. Neurology. (2017) 89:820–9. 10.1212/WNL.0000000000004259 28747441 PMC5580863

[12] Zhao Y Li X Zhang K Tong T Cui R. The Progress of Epilepsy after Stroke. Curr Neuropharmacol. (2018) 16:71–8. 10.2174/1570159X15666170613083253 28606039 PMC5771387

[13] Hendricks HT van Limbeek J Geurts AC Zwarts MJ. Motor recovery after stroke: a systematic review of the literature. Arch Phys Med Rehabil. (2002) 83:1629–37. 10.1053/apmr.2002.35473 12422337

[14] Shah SD Kalita J Misra UK Mandal SK Srivastava M. Prognostic predictors of thalamic hemorrhage. J Clin Neurosci. (2005) 12:559–61. 10.1016/j.jocn.2004.08.010 15936200

[15] Matsubara S Sato S Kodama T Egawa S Nakamoto H Toyoda K. Nonconvulsive status epilepticus in acute intracerebral hemorrhage. Stroke. (2018) 49:1759–61. 10.1161/STROKEAHA.118.021414 29880553

[16] Biffi A Rattani A Anderson CD Ayres AM Gurol EM Greenberg SM. Delayed seizures after intracerebral haemorrhage. Brain. (2016) 139:2694–705. 10.1093/brain/aww199 27497491 PMC5035821

[17] Aguilar MI Freeman WD. Spontaneous intracerebral hemorrhage. Semin Neurol. (2010) 30:555–64. 10.1055/s-0030-1268865 21207348

[18] van Asch CJ Luitse MJ Rinkel GJ van der Tweel I Algra A Klijn CJ. Incidence, case fatality, and functional outcome of intracerebral haemorrhage over time, according to age, sex, and ethnic origin: a systematic review and meta-analysis. Lancet Neurol. (2010) 9:167–76. 10.1016/S1474-4422(09)70340-0 20056489

[19] Chiu CC Li YN Lin LJ Hsiao CT Hsiao KY Chen IC. Serum D-dimer as a predictor of mortality in patients with acute spontaneous intracerebral hemorrhage. J Clin Neurosci. (2012) 19:810–3. 10.1016/j.jocn.2011.08.032 22377638

[20] Hu X Fang Y Ye F Lin S Li H You C. Effects of plasma D-dimer levels on early mortality and long-term functional outcome after spontaneous intracerebral hemorrhage. J Clin Neurosci. (2014) 21:1364–7. 10.1016/j.jocn.2013.11.030 24631325

[21] Delgado P Alvarez-Sabin J Abilleira S Santamarina E Purroy F Arenillas JF. Plasma d-dimer predicts poor outcome after acute intracerebral hemorrhage. Neurology. (2006) 67:94–8. 10.1212/01.wnl.0000223349.97278.e0 16832084

[22] Varju I Kolev K. Networks that stop the flow: A fresh look at fibrin and neutrophil extracellular traps. Thromb Res. (2019) 182:1–11. 10.1016/j.thromres.2019.08.003 31415922

[23] Longstaff C Varju I Sotonyi P Szabo L Krumrey M Hoell A. Mechanical stability and fibrinolytic resistance of clots containing fibrin, DNA, and histones. J Biol Chem. (2013) 288:6946–56. 10.1074/jbc.M112.404301 23293023 PMC3591605

[24] Wang F Zhang XY Hu FY Li FL Tian YC. Quantitative electroencephalography analysis for improved assessment of consciousness in cerebral hemorrhage and ischemic stroke patients. IEEE Access. (2019) 7:63674–85. 10.1109/ACCESS.2019.2916165

[25] Hirose G Saeki M Kosoegawa H Takado M Yamamoto T Tada A. Delta waves in the EEGs of patients with intracerebral hemorrhage. Arch Neurol. (1981) 38:170–5. 10.1001/archneur.1981.00510030064009 7469850

[26] Woo KM Yang SY Cho KT. Seizures after spontaneous intracerebral hemorrhage. J Korean Neurosurg Soc. (2012) 52:312–9. 10.3340/jkns.2012.52.4.312 23133718 PMC3488638

[27] Laccheo I Sonmezturk H Bhatt AB Tomycz L Shi Y Ringel M. Non-convulsive status epilepticus and non-convulsive seizures in neurological ICU patients. Neurocrit Care. (2015) 22:202–11. 10.1007/s12028-014-0070-0 25246236

[28] Hemphill JC 3rd Greenberg SM Anderson CS Becker K Bendok BR Cushman M. Guidelines for the management of spontaneous intracerebral hemorrhage: a guideline for healthcare professionals from the American heart association/American stroke association. Stroke. (2015) 46:2032–60. 10.1161/STR.0000000000000069 26022637

[29] Doria JW Forgacs PB. Incidence, implications, and management of seizures following ischemic and hemorrhagic stroke. Curr Neurol Neurosci Rep. (2019) 19:37. 10.1007/s11910-019-0957-4 31134438 PMC6746168

[30] Cortes M Black-Schaffer RM Edwards DJ. Transcranial magnetic stimulation as an investigative tool for motor dysfunction and recovery in stroke: an overview for neurorehabilitation clinicians. Neuromodulation. (2012) 15:316–25. 10.1111/j.1525-1403.2012.00459.x 22624621 PMC3760962

[31] Nagao S Kawai N. Prediction of motor function by magnetic brain stimulation in patients with intracerebral hematoma. Neurol Med Chir. (1992) 32:268–74. 10.2176/nmc.32.268 1378942

[32] Tsai SY Tchen PH Chen JD. The relation between motor evoked potential and clinical motor status in stroke patients. Electromyogr Clin Neurophysiol. (1992) 32:615–20. 1493776

